# Cocainising the Spinal Cord

**Published:** 1899-07-15

**Authors:** 


### COCAINISING THE SPINAL CORD.

AMONG recently suggested methods for the production of anæsthesia is the direct application of cocaine to the spinal cord by means of a "lumbar puncture." The whole proceeding is simplicity itself. After a preliminary local anæsthesia by Schleich's infiltration, a lumbar puncture is made, and a small quantity of a dilute solution of cocaine is injected. This is imagined to influence the spinal ganglia, the root zones, and the medullated fibres before they emerge from the cord, and thus to produce a complete analgesia below the line of injection. It is extremely probable that it does, but we think, at present, we would prefer to take ether.

